# Re-Parameterization After Pruning: Lightweight Algorithm Based on UAV Remote Sensing Target Detection

**DOI:** 10.3390/s24237711

**Published:** 2024-12-02

**Authors:** Yang Yang, Pinde Song, Yongchao Wang, Lijia Cao

**Affiliations:** 1 School of Automation & Information Engineering, Sichuan University of Science & Engineering, Yibin 644000, China; 322081104111@stu.suse.edu.cn (Y.Y.); tsingachieve@gmail.com (P.S.); 2 School of Aerospace Science and Technology, Xidian University, Xi’an 710071, China; ycwang9103@126.com; 3 Artificial Intelligence Key Laboratory of Sichuan Province, Yibin 644000, China; 4 Key Laboratory of Higher Education of Sichuan Province for Enterprise Informationalization and Internet of Things, Yibin 644000, China

**Keywords:** lightweight, object detection, re-parameterization, pruning, UAV remote sensing

## Abstract

Lightweight object detection algorithms play a paramount role in unmanned aerial vehicles (UAVs) remote sensing. However, UAV remote sensing requires target detection algorithms to have higher inference speeds and greater accuracy in detection. At present, most lightweight object detection algorithms have achieved fast inference speed, but their detection precision is not satisfactory. Consequently, this paper presents a refined iteration of the lightweight object detection algorithm to address the above issues. The MobileNetV3 based on the efficient channel attention (ECA) module is used as the backbone network of the model. In addition, the focal and efficient intersection over union (FocalEIoU) is used to improve the regression performance of the algorithm and reduce the false-negative rate. Furthermore, the entire model is pruned using the convolution kernel pruning method. After pruning, model parameters and floating-point operations (FLOPs) on VisDrone and DIOR datasets are reduced to 1.2 M and 1.5 M and 6.2 G and 6.5 G, respectively. The pruned model achieves 49 frames per second (FPS) and 44 FPS inference speeds on Jetson AGX Xavier for VisDrone and DIOR datasets, respectively. To fully exploit the performance of the pruned model, a plug-and-play structural re-parameterization fine-tuning method is proposed. The experimental results show that this fine-tuned method improves mAP@0.5 and mAP@0.5:0.95 by 0.4% on the VisDrone dataset and increases mAP@0.5:0.95 by 0.5% on the DIOR dataset. The proposed algorithm outperforms other mainstream lightweight object detection algorithms (except for FLOPs higher than SSDLite and mAP@0.5 Below YOLOv7 Tiny) in terms of parameters, FLOPs, mAP@0.5, and mAP@0.5:0.95. Furthermore, practical validation tests have also demonstrated that the proposed algorithm significantly reduces instances of missed detection and duplicate detection.

## 1. Introduction

Neural network-based object detection methods are essential in various application scenarios, including face detection [1], traffic sign detection [2], vehicle detection [3], remote sensing images [4,5,6], and unmanned aerial vehicles (UAVs) remote sensing [7,8,9,10]. Especially on edge computing devices with limited computing power carried by UAVs, detection algorithms for UAVs and remote sensing tasks require higher real-time performance and accuracy.

Contemporary object detection techniques leveraging convolutional neural networks (CNNs) can be broadly classified into two primary categories: dual-phase and single-phase detection algorithms. These approaches represent cutting-edge methodologies in the field of automated visual object recognition. Over the past decade, several two-stage object detection algorithms have been proposed, including Region-CNN (R-CNN) [11], Faster R-CNN [12], and Mask R-CNN [13]. However, these methods tend to have intricate network structures and are not lightweight, which can hinder their deployment in real-time applications and embedded devices. In contrast, the one-stage object detection method, for instance, you only look one (YOLO) [14] and Single-Shot Detector (SSD) [15], achieves an improved equilibrium between computational efficiency and recognition precision. YOLO, in particular, has emerged as a prevalent algorithm extensively employed in diverse downstream detection tasks. Despite significant efforts in developing SOTA algorithms such as YOLO9000 [16], YOLOv3 [17], YOLOv4 [18], YOLOv5, and YOLOX [19], these algorithms still require substantial computational resources to achieve high accuracy and faster inference speed, resulting in significant hurdles for real-world integration in real-time on edge computing devices. To address this challenge, researchers have undertaken comprehensive investigations into lightweight object detection algorithms, aiming to develop more lightweight models while maintaining satisfactory detection performance.

There are two commonly used methods to reduce the parameters of a detection algorithm: engineering compact architectures through backbone network substitution or developing lighter architectures, and model pruning.

Huang et al. [20] devised a detection method based on YOLO9000 by redesigning its network architecture, specifically aimed at efficient operation on non-GPU devices. Redmon et al. proposed YOLOv3-Tiny based on YOLOv3, which reduces the number of layers and detection heads, resulting in improved inference performance compared to YOLOv3. Wong et al. [21] proposed YOLO leveraged depth-wise separable convolutions in its network architecture design. Compared to YOLOv2-Tiny and YOLOv3-Tiny, YOLO Nano achieves higher detection accuracy with lower parameters. Bochkovskiy et al. [18] proposed YOLOv4 and also released YOLOv4-Tiny, a lightweight variant. Another significant contribution is YOLOv5, proposed by the Ultralytics company. YOLOv5 adopts a parameter control mechanism and manages the total of parameters and floating-point operations (FLOPs) through width and depth parameters. This approach enables fine-grained control over the computational efficiency of the neural architecture. Two lightweight versions, YOLOv5s and YOLOv5n, have been released. Moreover, several other notable YOLO algorithms have also released their respective lightweight versions. For example, YOLOv6n [22], YOLOv7-Tiny [23], YOLOX-Tiny, and YOLOv8s have all proposed lightweight detectors by directly designing simple network models. A balance between parameter parsimony and detection prowess is achieved in these models.

As lightweight networks evolve at an unprecedented pace, there is an increasing adoption of lightweight backbone networks in object detection algorithms. For example, Sandler et al. [24] devised a minimally resource-intensive object method based on MobileNetV2 and SSD. Zhang et al. [25] devised the ShuffleNet lightweight model and used it as the backbone network of the Faster-RCNN. Howard et al. [26] devised MobileNetV3 and used it as a drop-in replacement for the backbone feature extractor in SSDLite. Han et al. [27] proposed the GhostNet and applied it to RetinaNet. These backbone networks have significantly reduced the parameters and FLOPs of object detection algorithms, striking an optimal equilibrium between precision in detection and computational efficiency. Sun et al. [28] proposed a lightweight dual Siamese network (SiamOHOT) for airborne hyperspectral target tracking.

Both directly designed object detection algorithms and improved object detection algorithms based on lightweight backbone networks have showcased remarkable inference performance. However, ensuring the accuracy of these object detection algorithms poses a challenge due to the constrained number of variables in their network and the limited content they can learn.

Weight pruning techniques have been demonstrated as one of the most effective strategies to decrease intensive computations and memory requirements while preserving model accuracy. By eliminating redundant weights, models with structural sparsity improved memory and power efficiency, as well as reduced inference latency. Notably, Li et al. [29] successfully addressed the issue of large parameters in YOLOv3 through pruning, which, through innovative design choices, achieved a leaner parameter of YOLOv3 by three times. Wu et al. [30] applied a channel pruning method to prune the backbone network of YOLOv4, achieving a remarkable 96.7% reduction in parameters. Zhang et al. [31] employed pruning on YOLOv5l, resulting in a significant reduction of 63.8% in parameters and 37.4% in FLOPs, striking a favorable parity between computational resource utilization and detection exactitude. Additionally, Gupta et al. [32] proposed a pruning fine-tuning algorithm for YOLOv6, which boosted the frame per second (FPS) of the pruned model by 1.9 times compared to the YOLOv6 baseline.

The aforementioned methods have made significant contributions to lightweight object detection algorithms. However, the following problems remain:

(1) Redundant parameters: Despite employing lightweight models as backbone networks for object detection algorithms, there remains a substantial number of redundant parameters within the detector model. This redundancy hampers the overall efficiency of the detection algorithm.

(2) Performance exploration: While fine-tuning can restore the performance of pruned models, further enhancements in network learning capability can be achieved by optimizing the pruned models before fine-tuning. This allows for an improved equilibrium between predictive performance and computational efficiency.

In an effort to thoroughly investigate the efficacy of the streamlined model and attain an optimal equilibrium between detection fidelity and processing velocity, we proposed a lightweight version of the object detection model, PR-YOLO, which is based on the YOLOv5m architecture. The primary advancements put forth by this study can be summarized as follows:

(1) To address the issue of redundant parameters mentioned above, the MobileNetV3 improved with the efficient channel attention (ECA) [33] mechanism module is introduced into the backbone network of YOLOv5m. This replaces the lightweight backbone network to reduce the number of model parameters.

(2) For the problem of air-to-ground target detection, focal and efficient intersection over union (FocalEIoU) [34] is used as the regression loss function for bounding boxes to optimize the model’s localization capability during training.

(3) To further explore improvements in model performance, an optimization pruning fine-tuning algorithm based on reparameterization is proposed to further enhance the performance of the fine-tuned model. Specifically, before fine-tuning the pruned model, branch expansion was performed on the pruned model (i.e., converting a single branch into multiple branches). Once the model regains its accuracy through fine-tuning, the multiple branches are merged back into a single branch using structural re-parameterization to further enhance the model’s performance.

The subsequent sections of this manuscript are structured in the following manner. Section 2 introduces the related work. Section 3 provides details of our proposed PR-YOLO and a pruning fine-tuning method based on re-parameterization. Section 4 discusses the experimental implementation, results, and comparison with other mainstream lightweight algorithms. Section 5 is the discussion. The conclusion of our work is in Section 6.

## 2. Materials

### 2.1. Lightweight CNN Models

Lightweight models with fewer parameters and lower computational complexity have been developed rapidly. This development can be traced back to the proposed SqueezeNet by Iandola et al. [35] in 2016, which addressed the issue of large network parameters in AlexNet. MobileNetV1’s framework leverages a combination of depth-wise and point-wise convolutional layers to significantly reduce the computational complexity of the model, resulting in improved network inference speed [36]. ShuffleNet addresses the problem of irrelevant channel information in depth-wise convolution by employing channel shuffle. MobileNetV2 takes this further by introducing residual connections to the lightweight model and constructing an inverted residual structure, leading to higher accuracy and faster inference speed [24]. Tan et al. [37] and Howard et al. [26] devised MnasNet and MobileNetV3, which are lightweight models that have been developed through the utilization of neural architecture search (NAS). These models demonstrate excellent performance with respect to both speed and precision. Han et al. [27] devised the lightweight GhostNet by analyzing redundant information in the feature maps and replacing half of the convolution channels with cheaper operations. So far, lightweight networks such as GhostNetV2 [38], MobileOne [39], and FasterNet [40] have achieved good performance in object detection tasks.

### 2.2. Model Pruning

Pruning methods play a crucial role in reducing redundant parameters within networks, and they can be categorized into unstructured pruning and structured pruning.

Unstructured pruning can obtain higher pruning rates, and any weight in the model can be pruned. However, unstructured pruning can result in irregular sparsity in the weight matrix, which is difficult to accelerate by general hardware acceleration. Conversely, the application of unstructured pruning maintains the network’s depth and width intact. Therefore, the acceleration effect of the pruned model using this method is limited.

Structured pruning prunes the entire channels or filters of weights. The filter pruning removes the whole filter of the weight matrix, while channel pruning removes channels with identical sequences in different filters. Therefore, structured pruning can effectively compress network parameters, thereby improving the inference speed of the model.

### 2.3. Model Re-Parameterization

The idea of re-parameterization is first proposed by Zagoruyko et al. [41] in DiracNets. The model removes skip connections by changing the kernel weight in ResBlock. It can be defined as $\hat{W}=diag\left(a\right)W_{norm}+diag\left(b\right)I$, where $\hat{W}$ is the merged convolutional kernel weight, a and b are learnable variables, $W_{norm}$ is the weight of the convolutional kernel before merging. DiracNets provides an important approach for re-parameterization, but this method does not gain in model performance.

**Figure 1:**
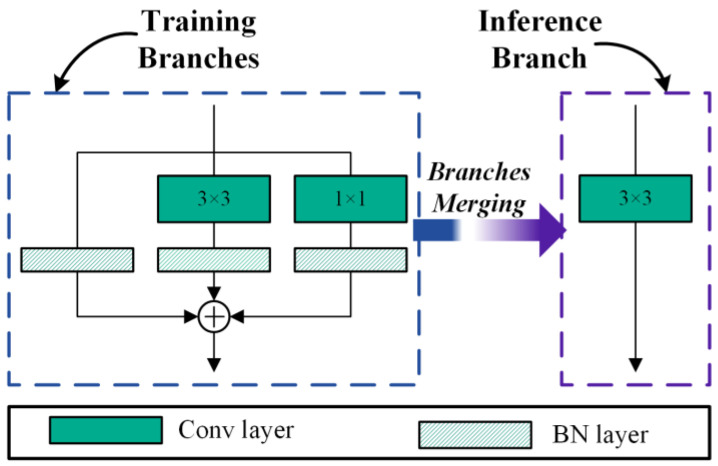
Structural re-parameterization of a RepVGG module.

Zhang et al. [25] proposed an efficient inference rule for networks: the fewer branches a model has, the faster its speed. Therefore, single-branch networks are often used in embedded devices with limited computing resources. Although single-branch networks have excellent inference performance, their learning capability is not as good as multi-branch networks. How can we address the problem if we want the network to have excellent performance and efficient inference speed? RepVGG [42] provides a solution method. It builds a training model using a multi-branch during training and then converts the multi-branch to a single branch during inference. The principle of RepVGG is presented in Figure 1.

As shown in Figure 1, there are three branches during the training process. The $W^{\left(3\right)}\in {\mathbb{R}}^{C_{out}\times C_{in}\times 3\times 3}$ denotes the weight of a 3×3 conv layer, and the $W^{\left(1\right)}\in {\mathbb{R}}^{C_{out}\times C_{in}\times 1\times 1}$ denotes the weight of 1×1 conv layer, where $C_{in}$ is the input channels and $C_{out}$ is the output channels. In the BN layer, $\beta^{\left(k\right)}$ denote bias, $\gamma^{\left(k\right)}$ denote learned scaling factor, $\mu^{\left(k\right)}$ denote accumulated mean, $\sigma^{\left(k\right)}$ denote standard deviation, where k for the convolutional kernel size. Suppose the size of the input feature map is $F^{\left(in\right)}\in {\mathbb{R}}^{N\times C_{in}\times H_{in}\times W_{in}}$, and the size of the output feature map is $F^{\left(out\right)}\in {\mathbb{R}}^{N\times C_{out}\times H_{out}\times W_{out}}$; ∗ is the convolution operator. If $C_{in}=C_{out}$,$W_{in}=W_{out}$,$H_{in}=H_{out}$, we have the following:

(1) $$F^{\left(out\right)}=BN\left(F^{\left(in\right)},\mu^{\left(0\right)}\sigma^{\left(0\right)},\gamma^{\left(0\right)},\beta^{\left(0\right)}\right)+\sum_{k=1,3}BN\left(F^{\left(in\right)}*W^{\left(k\right)},\mu^{\left(k\right)}\sigma^{\left(k\right)},\gamma^{\left(k\right)},\beta^{\left(k\right)}\right)$$

Upon completion of the model’s training phase, a crucial step involves merging the convolution and batch normalization layers. The mathematical formulation of the Batch Normalization BN process can be expressed as follows:

(2) $$BN\left(F,\mu ,\sigma ,\gamma ,\beta \right)=\left(F-\mu \right)\frac{\gamma }{\sigma }+\beta$$

The convolution operator without bias can be defined as follows:

(3) $$F=F^{\left(in\right)}*W^{\prime }$$

where $W^{\prime }$ is the weight of the convolution kernel. The fuse result can be computed as follows:

(4) $$BN\left(F,\mu ,\sigma ,\gamma ,\beta \right)=F^{\left(in\right)}*W^{\prime }\frac{\gamma }{\sigma }+\left(\beta -\mu \frac{\gamma }{\sigma }\right)$$

Then it is easy to simplify Equation (1). It can be simplified as follows:

(5) $$F^{\left(out\right)}=F^{\left(in\right)}*\sum_{k=0,1,3}W^{\left(k\right)}\frac{\gamma^{\left(k\right)}}{\sigma^{\left(k\right)}}+\sum_{k=0,1,3}\left(\beta^{\left(k\right)}-\mu^{\left(k\right)}\frac{\gamma^{\left(k\right)}}{\sigma^{\left(k\right)}}\right)=F^{\left(in\right)}*W^{\left(3\right)}+b^{\left(3\right)}$$

where $W^{\left(k\right)}$ indicates the weight of convolutional kernel, $W^{\left(3\right)}$ indicates the weight of convolution kernel after merging multi-branch, and $b^{\left(3\right)}$ indicates the bias of convolution kernel. The merged result is the single branch in Figure 1.

## 3. Methods

### 3.1. Proposed the PR-YOLO Model

**Figure 2:**
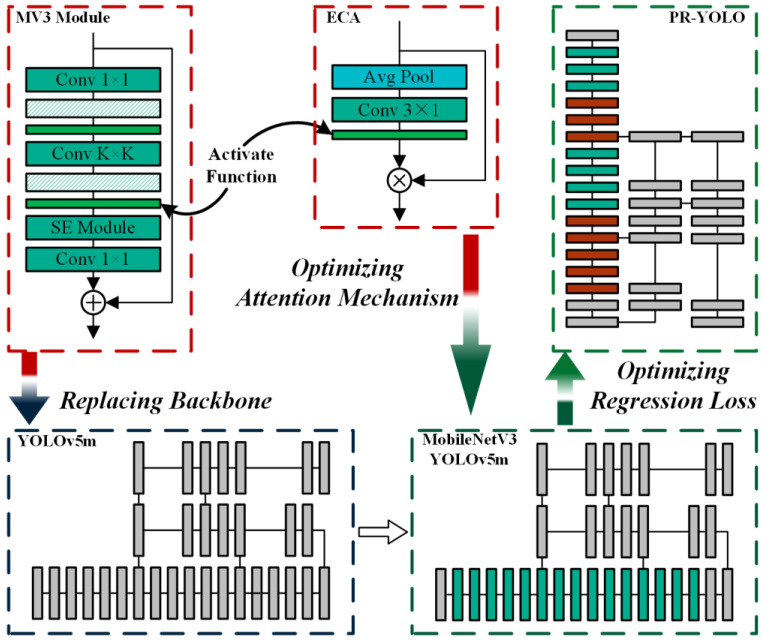
The architecture of the PR-YOLO.

Striving for an optimal equilibrium between detection capability and processing celerity, a computationally efficient detection method based on YOLOv5m is proposed. Figure 2 shows the improved YOLOv5m structure. The improved MobileNetV3, replacing the squeeze and excitation (SE) module by ECA, is integrated into the YOLOv5m backbone network. In addition, the FocalEIoU as the bonding box regression loss function is also introduced into the YOLOv5m. The proposed ECA module is characterized by its minimal parameter footprint and substantial performance enhancement (better accuracy and less inference time). The purpose of FocalEIoU is to enhance the regression capability.

Except for the modules already labeled in Figure 2, the green diagonal-striped modules represent BN, the bright green modules represent the SilU activation function, and the gray modules are the unmodified YOLOv5m.

#### 3.1.1. Backbone Network Improvement

**Figure 3:**
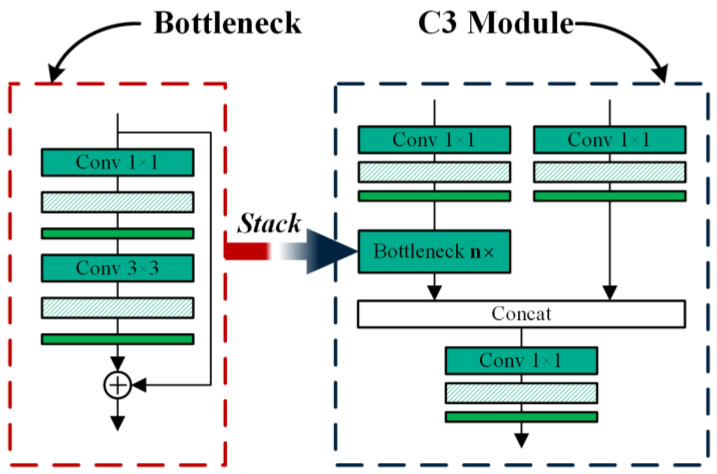
The structure of the C3 module.

The MV3 module is proposed in MobileNetV3. It uses the inverted residual structure to extract features and introduces the SE attention module to enhance inter-channel information exchange. Compared to the C3 module of YOLOv5, its parameters and computational cost are relatively low. The structure of the C3 module and the MV3 module are shown in Figure 2 and Figure 3.

The architectural configuration of the C3 module is delineated in Figure 3. Suppose the size input feature map is $I\in {\mathbb{R}}^{c\times h\times w}$, the dimensions of the resultant feature map is $O\in {\mathbb{R}}^{c\times h\times w}$, and the quantity of stack is n, where h is the height of the feature map, w is the width of the feature map, and c is the channel quantity of input and output feature maps. Excluding the batch normalization layer parameters, we can compute the C3 module’s parameter count as follows:

(6) $$P_{C3}=c\times \frac{c}{2}+c\times \frac{c}{2}+c\times c+\frac{c}{2}\times \frac{c}{2}\times n+3\times 3\times \frac{c}{2}\times \frac{c}{2}\times n=\left(2.5n+2\right)c^{2}$$

The computational complexity of the C3 module, expressed in FLOPs, can be derived using the following method:

(7) $$F_{C3}=2h\times w\times c\times c+2.5n\times h\times w\times c\times c=\left(2.5n+2\right)hwc^{2}$$

From Figure 3, ignoring the SE module and BN layer, the parameters of the MV3 component can be computed as follows:

(8) $$P_{d}=c\times e+k\times k\times e+e\times c=2ce+ek^{2}$$

where k indicates the dimensions of convolution kernel, e indicates hidden channel.

In an analogous manner, we can derive the computational complexity of the MV3 component, expressed in FLOPs, as follows:

(9) $$F_{d}=2h\times w\times c\times e+k\times k\times h\times w\times e=\left(2c+k^{2}\right)ehw$$

Comparing the C3 component with the parameters, and the FLOPs of the MV3 component, The analytical expression for the theoretical ratio is determined as follows:

(10) $$r=\frac{P_{C3}}{P_{d}}=\frac{\left(2.5n+2\right)c^{2}}{\left(2c+k^{2}\right)e}$$

In the MV3 module, the minimum ratio of the output channel to the hidden channel is approximately 0.17 and the maximum ratio is 1.0. When the channel ratio is 0.17, we set n to 6. Similarly, when the channel ratio is 1.0, n is 1. If k=3 and c=64, we have the following:

(11) 1.2<r<2.1

According to the Equation (11), swapping the C3 component with the MV3 component can effectively mitigate the overall parameters and FLOPs of the component.

In MobileNetV3, the SE component is used in some of the MV3 components to enhance the focus on the channel information of the algorithm. Although the SE component enhances the performance of the algorithm, the component is not lightweight. In addition, convolutional layers are the most time-consuming part during both training and inference processes. Therefore, reducing the number of convolutional layers constitutes a primary determinant of optimizing the inference performance of model. As illustrated in Figure 2, the SE component in MobileNetV3 is exchanged for the ECA component to reduce the number of convolution layers. The ECA module can be calculated as follows:

(12) $$F=\sigma \left(W_{e}*F_{avg}\right)\times F_{in}$$

where $F_{in}$ denotes input feature maps, $F_{avg}$ denotes the features of average pooling, $W_{e}$ indicates the weight of convolution kernel, σ denotes the sigmoid function, F denotes the output feature maps.

#### 3.1.2. Regression Loss Function Improvement

The function utilized for quantifying bounding box regression error is defined as the CIoU in YOLOv5. It can be defined as follows:

(13) $$\begin{array}{l} L_{CIoU}=1-IoU+\frac{\rho^{2}\left(b,b^{gt}\right)}{c^{2}}+\alpha \nu \\ \nu =\frac{4}{\pi^{2}}{\left(arctan\frac{\omega^{gt}}{h^{gt}}-arctan\frac{\omega }{h}\right)}^{2}\\ \alpha =\frac{v}{\left(1-IoU\right)+v} \end{array}$$

where α stands for the weighting constant, ρ for the Euclidean distance, ν for the proportional correspondence of dimensions between forecasted and actual results, c for the Euclidean distance along the diagonal axis between the hypothesized and actual bounding boxes, $b^{gt}$ for the spatial data of reference bounding boxes, b for the spatial data of estimated object regions, and intersection over union (IOU) is the intersection percentage of reference bounding area and prediction result.

The Complete Intersection over Union (CIoU) metric enhances traditional IOU by incorporating an aspect ratio penalty term. This addition aims to minimize the shape discrepancy between model-generated zones and validated target areas by accounting for their dimensional differences. However, by analyzing the derivative of ν, it is found that w and h cannot increase or decrease simultaneously, which leads to the inefficiency issue of the spatial boundary adjustment process.

Due to the limitation of the CIoU, which only penalizes the relative aspect ratios and fails to consider the size variations between reference and model-generated bounding areas, as well as the imbalance issue of hard positive and negative samples. Given its effectiveness, FocalEIoU has been selected as the regression loss metric for the object detection task outlined in this research. The FocalEIoU can be defined as follows:

(14) $$\begin{array}{l} L_{EIoU}=1-IoU+\frac{\rho \left(b,b^{gt}\right)}{c^{2}}+\frac{\rho \left(w,w^{gt}\right)}{c_{w}^{2}}+\frac{\rho \left(h,h^{gt}\right)}{c_{h}^{2}}\\ L_{FocalEIoU}=IoU^{\gamma }L_{EIoU} \end{array}$$

where $c_{w}$ and $c_{h}$ indicate the horizontal and vertical extents of the minimum enclosing rectangle that contains both bounding boxes, w and $w^{gt}$ indicate the width of the ground truth box and prediction box, h and $h^{gt}$ indicate the height of the ground truth box and prediction box, γ denotes a hyperparameter.

### 3.2. Re-Parameterization Towards the Larger Kernel

**Figure 4:**
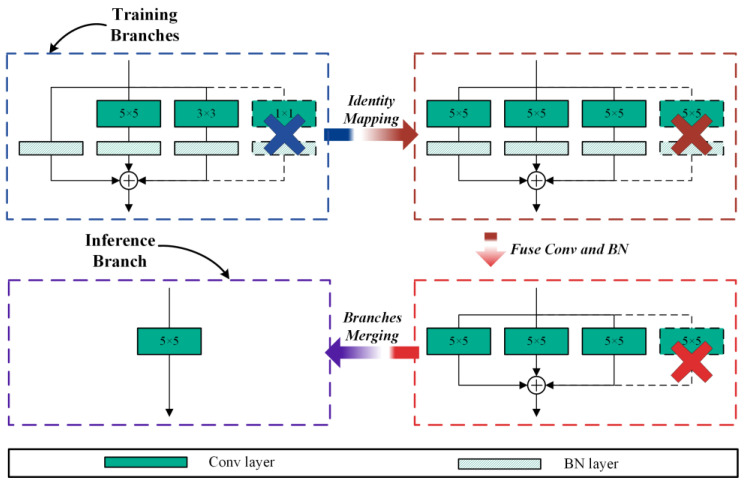
The training and inference structure of 5 × 5 convolutional branches.

**Figure 5:**
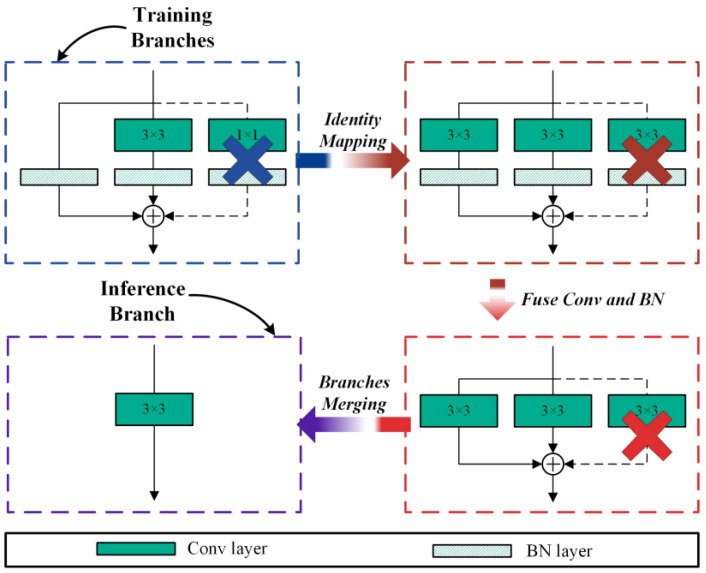
The training and inference structure of 3 × 3 convolutional branches.

In recent years, structural re-parameterization methods have been widely applied in backbone network design. Network models such as MobileOne, RepGhost [43], and RepVGG have utilized structural re-parameterization methods to enhance model performance. However, during the training process, these models primarily focused on the impact of 1 × 1 convolution branches and BN branches on model performance, while overlooking the potential impact of larger convolution kernels on model performance. Taking the RepGhost as an example, despite sharing consistent design paradigms in network structure with MobileNetV3 and utilizing larger convolutional kernels (5 × 5). RepGhost, when optimizing GhostNet using structural re-parameterization, does not consider the performance impact of the 3 × 3 convolution branch on the 5 × 5 convolution branch. To address the aforementioned issue, this study focuses on the MobileNetV3 backbone network and utilizes structural re-parameterization to optimize the network branches, endeavoring to further ameliorate the effectiveness of the model. Figure 4 and Figure 5 show the structure of the MV3 module in the course of the training and inference process.

Figure 4 illustrates that throughout the model’s training iteration, the 5 × 5 convolution branch in the MobileNetV3 backbone network is expanded into parallel 5 × 5, 3 × 3, and BN branches. However, during the inference phase, all the parallel branches are merged into a single 5 × 5 branch. The transformation method of the 3 × 3 convolution branch in Figure 5 is the same as that in Figure 4. The black dashed lines and X in Figure 4 and Figure 5 indicate that the branch has been deleted.

Taking Figure 4 as an example, suppose that the input, the output, the number of channels, and BN layer parameters are consistent with those in Figure 1. The calculation formulas for training branches are as follows:

(15) $$F^{\left(out\right)}=BN\left(F^{\left(in\right)},\mu^{\left(0\right)}\sigma^{\left(0\right)},\gamma^{\left(0\right)},\beta^{\left(0\right)}\right)+\sum_{k=3,5}BN\left(F^{\left(in\right)}*W^{\left(k\right)},\mu^{\left(k\right)}\sigma^{\left(k\right)},\gamma^{\left(k\right)},\beta^{\left(k\right)}\right)$$

According to Equations (2)–(4), the inference branch can be computed as follows:

(16) $$F^{\left(out\right)}=F^{\left(in\right)}*\sum_{k=0,3,5}W^{\left(k\right)}\frac{\gamma^{\left(k\right)}}{\sigma^{\left(k\right)}}+\sum_{k=0,3,5}\left(\beta^{\left(k\right)}-\mu^{\left(k\right)}\frac{\gamma^{\left(k\right)}}{\sigma^{\left(k\right)}}\right)=F^{\left(in\right)}*W^{\left(5\right)}+b^{\left(5\right)}$$

where $W^{\left(5\right)}$ denotes the numerical parameters defining the convolution operator, $b^{\left(5\right)}$ denotes the bias of the convolution kernel.

Compared to RepVGG, MobileOne, and RepGhost, the 1 × 1 convolution branch is not used in our proposed structural re-parameterization of MV3 module. Why do not use the 1 × 1 convolution branch? Through experiments, it was found that the 1 × 1 convolution branch is useless and can even reduce model performance when the main branch is depth-wise convolution.

### 3.3. Pruning and Fine-Tuning Process Improvement

**Figure 6:**
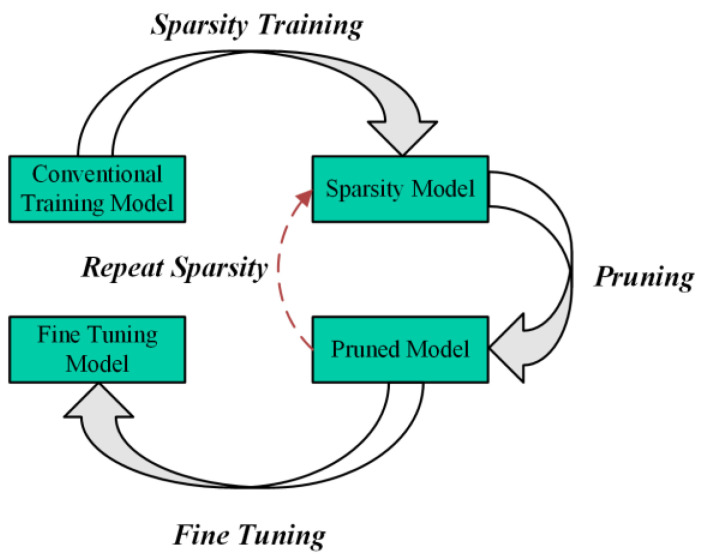
Original pruning and fine-tuning process.

Structured pruning is commonly used to compress the width of a model. Liu et al. [44] introduced a scaling factor γ for each channel and performed sparsity training on the network, pruning unimportant convolution kernels and output channels. After completing the model pruning, the fine-tuning method is applied to further restore the predictive precision of the algorithm, thus achieving the entire pruning process. The sequential stages of neural network dimensionality reduction are delineated in Figure 6.

**Figure 7:**
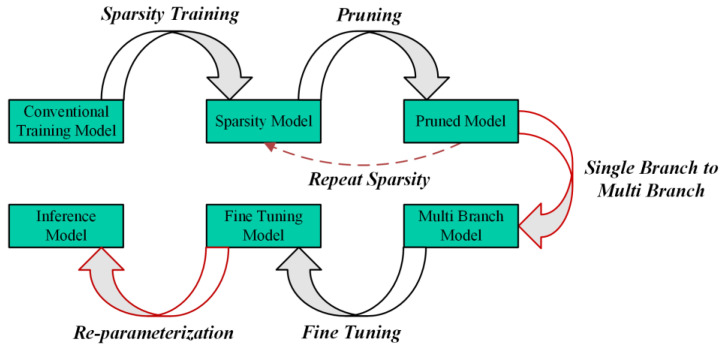
Improved pruning and fine-tuning process.

After pruning, the number of parameters in the pruned model often drops dramatically, precipitating a decline in the system’s performance metrics. Then, fine-tuning can be used to further restore the accuracy of the model; it can even achieve higher accuracy than the model before pruning. Generally, the pruned model has fewer parameters and FLOPs, which means that the learning and generalization ability of the network is reduced. Given this challenge, we are considering whether there is an approach to further boost the learning capacity of the model so that the pruned model can achieve higher accuracy through fine-tuning. Inspired by the approach of structural re-parameterization, we propose an enhanced process of pruning and fine-tuning, further investigating the performance of a lightweight model. The enhanced pruning and fine-tuning process is illustrated in Figure 7.

As illustrated in Figure 7, we convert the single branch of the MV3 module in the pruned MF-YOLO model into a multi-branch before fine-tuning. When the model recovers accuracy through fine-tuning, structural re-parameterization is used to transform a multi-branch structure into a single branch. Figure 4 illustrates the specific conversion process.

## 4. Experiments

To assess the performance of the devised solution, two datasets, namely the DIOR dataset and the VisDrone dataset, are utilized in the experiment. This section presents a comprehensive description of the algorithm implementation, training settings, and the experimental results.

### 4.1. Experimental Details

The devised algorithm is operationalized by the PyTorch deep learning framework, and the running environment of the algorithm is based on the Ubuntu 18.04 operating system. The test of the embedded platform is based on Jetson AGX Xavier. Table 1 displays the hardware devices used for training and testing.

The software version is Python3.6.15, CUDA10.2, and CUDNN8.4.0 in the training server. In Jetson AGX Xavier, the Jetpack version is 4.6.5.

The YOLOv5 is an object detection algorithm, and it is based on anchor boxes. Therefore, prior to training on a new model, it is essential to perform anchor box clustering on the dataset. In YOLOv5, the algorithm utilizes *k*-means to cluster the anchor boxes in the dataset and then employs a genetic algorithm to mutate the width and height of the anchor boxes in order to obtain optimal results. In this study, the anchor box for the VisDrone [43] and DIOR [44] datasets were computed using the k-means clustering algorithm. However, for the VisDrone dataset, the generated anchor boxes were not subjected to mutation using a genetic algorithm due to inferior results. The anchor box configurations for both the VisDrone and DIOR datasets are shown in Table 2.

The model’s training process comprises three stages.: conventional training, sparsity training, and fine-tuning training. In the conventional training stage, the linear learning rate is employed in the training process of the model. In the fine-tuning stage, a one-cycle learning rate is used to train the model. The linear learning rate function and the one-cycle learning rate can be defined as Equations (17) and (18). The hyperparameters of different training stages are shown in Table 3.

(17) $$L_{linear}=\left(1-\frac{e}{300}\right)\times \left(1-l_{f}\right)+l_{f}$$

(18) $$L_{one-cycle}=0.5\times \left(l_{f}-1\right)\left(1-cos\left(\frac{e\pi }{300}\right)\right)+1$$

where e denotes current epoch, $l_{f}$ denotes the final learning rate.

#### Evaluating Indicators

To assess the effectiveness of the suggested technique, several indicators are utilized as measurement indicators. These indicators include precision, recall, mAP@0.5, mAP@0.5:0.95, inference time, the count of parameters, FLOPs, and FPS. The precision can be defined as follows:

(19) $$P=\frac{TP}{TP+FP}$$

where TP stands for the total of correct positive specimens, and FP for the number of incorrect positive specimens.

Recall is the proportion of correctly identified positive specimens to the total positive specimens within a specific class. It can be defined as follows:

(20) $$R=\frac{TP}{TP+FN}$$

where FN denotes undetected positive samples.

The AP is a metric that quantifies the region under the precision-recall trajectory and is used to assess the overall effectiveness of each category in a detection task. Conversely, mAP computes the arithmetic mean of all AP values throughout various classifications. The formulas for calculating AP and mAP are as follows:

(21) $$AP=\int_{0}^{1}P\left(R\right)dR$$

(22) $$mAP=\frac{\sum_{i=1}^{N}AP_{i}}{N}$$

According to the defined mAP, when the IOU threshold is 0.5, the mAP is equal to mAP@0.5. When the IOU varies from 0.5 to 0.95 with a step size of 0.5, the mAP is identified as mAP@0.5:0.95.

FPS represents the total of images that can be detected in a second. The testing of these algorithms is based on Jetson AGX Xavier.

### 4.2. Training and Validation Datasets

Edge computing devices have extensive applications in various domains such as autonomous driving, UAV inspections, and telemetry. Therefore, this study evaluates the performance of the proposed method in UAVs and remote sensing scenes using the VisDrone and the DIOR datasets. The VisDrone dataset comprises 10 categories, containing 6471 images for training and 548 for validation. It ranks as one of the most extensively utilized datasets for detecting objects in UAV imagery. Meanwhile, the DIOR dataset acts as a comprehensive benchmark for detecting objects in optical remote sensing images. The dataset comprises 5862 training images and 5863 validation images, covering 20 object classes. Both datasets share a common characteristic: they contain a significant number of small object instances.

### 4.3. Experimental Results

#### 4.3.1. Ablation Experiments

The design of ablation experiments is used to verify the contribution of each module to the network. The results are shown in Table 4.

In Table 4, it is observed that when MobileNetV3 is employed as the backbone network for YOLOv5m, the total of parameters and FLOPs are decreased by 43.1% and 58.3%, correspondingly. As a result, the inference efficiency on the Jetson AGX Xavier device experiences a notable improvement of 8 FPS and 10 FPS, respectively. The ECA attention mechanism decreases the model’s parameters by 1.5 M while also slightly improving recognition accuracy. By swapping out the CIoU loss function for FocalEIoU, the proposed algorithm achieved higher mAP@0.5:0.95 on the VisDrone and DIOR datasets, with respective improvements of 0.6% and 0.9%. Specifically, the mAP@0.5:0.95 reached 19.3% for VisDrone and 57.5% for DIOR. When pruning the improved model, the total parameters and FLOPs of the model on the VisDrone dataset and DIOR dataset are 1.2 Mand 1.5 M, and 6.2 G and 6.5 G, respectively. After fine-tuning, the model achieves a mAP@0.5 of 35.8% and 79.8%, and a mAP@0.5:0.95 of 20.3% and 58.1% on the respective datasets. Notably, the pruned model achieves a significant increase in detection speed, with improvements of 9 FPS and 17 FPS on the VisDrone and DIOR datasets compared to the pre-pruned model, respectively. Furthermore, when applying the structural re-parameterization fine-tuning method to refine the models, the fine-tuned models demonstrate improvements of 0.4% in mAP@0.5 and 0.4% in mAP@0.5:0.95 on the VisDrone dataset and 0.5% in mAP@0.5:0.95 on the DIOR dataset. Although the proposed method did not achieve the identical level of detection precision as YOLOv5m, it demonstrates significant enhancements in inference efficiency, providing new insights for fine-tuning the model and exploring the performance of lightweight models.

#### 4.3.2. Model Compression Experiment

**Figure 8:**
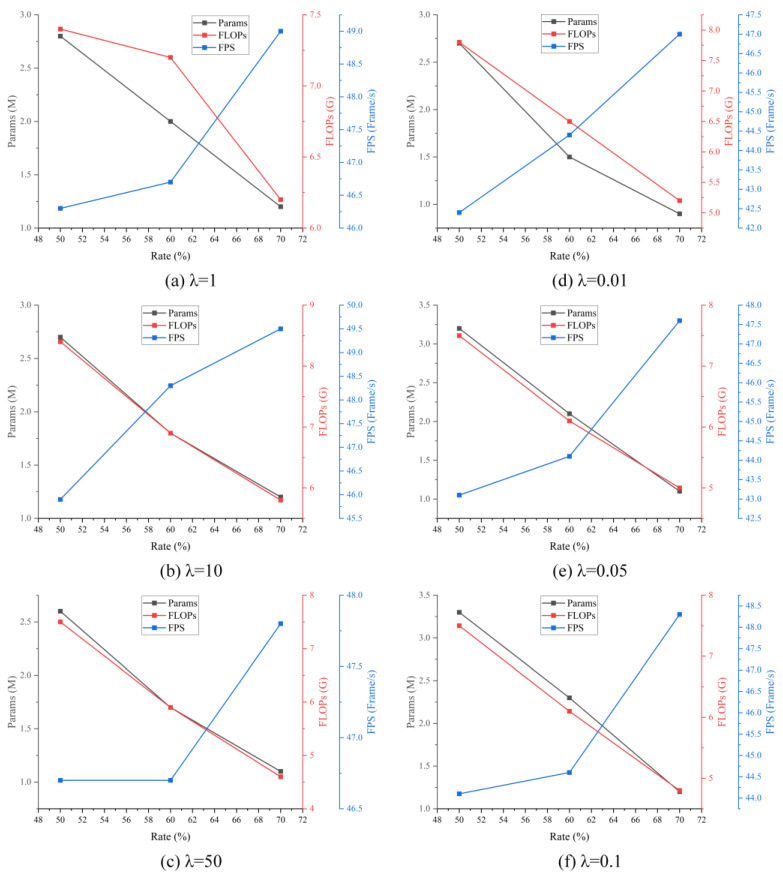
The results of the impact of pruning on model performance under different sparsity parameters.

The model compression evaluation is conducted based on the improved model in Section 4.3.1. Different sparsity constants are utilized during the training stage of the model to obtain the optimal sparsity model. For the VisDrone dataset, the sparsity constants are set to 1, 10, and 50, making a gradual approach of the parameters in the BN layer to zero. Similarly, for the DIOR, the sparsity constants are set to 0.01, 0.05, and 0.1, achieving the same effect. After completing the sparsity training, pruning is applied to the sparsity model with pruning rates of 50%, 60%, and 70%. The variations of parameters, FLOPs, and FPS with model compression under different sparsity constants and pruning rates are shown in Figure 8.

In Figure 8, regardless of the sparsity constant’s value, it’s clear that as the pruning rate increases, both the parameters and FLOPs are reduced. Additionally, there is an improvement in inference performance as the model parameters decrease. Specifically, with the pruning rate at 70%, the parameters of the model can be compressed to approximately 1 M from an initial value of 10 M, while the FLOPs decrease from 20 G to around 5–6 G. Moreover, the pruned model achieves an FPS close to 50. Comparing these results to the improved model in Table 4, both the parameters and FLOPs have been reduced by approximately 90% and 75%, respectively. Furthermore, the FPS increased by approximately 23 FPS and 11 FPS on the VisDrone and DIOR datasets, respectively. Consequently, the pruned model can run more efficiently on edge computing devices carried by UAVs.

#### 4.3.3. Fine-Tuning and Re-Parameterization Experiment

**Figure 9:**
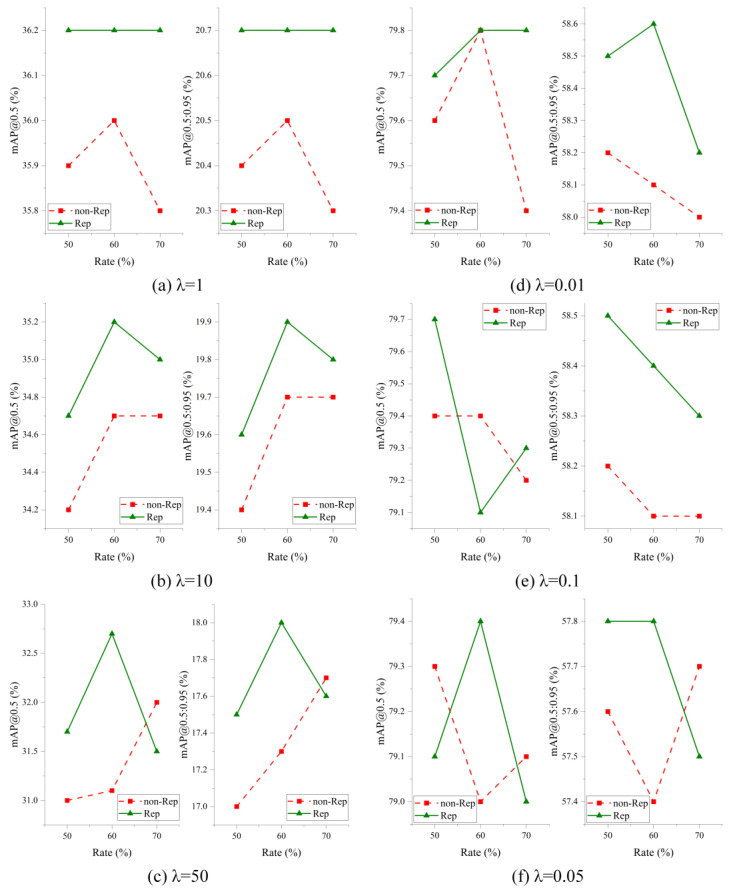
The results of structural re-parameterization on pruned models during fine-tuning.

To evaluate the influence of structural re-parameterization on the precision of pruned models during the fine-tuning process, this study performs rigorous verification and analysis. Different sparsity constants and pruning rates are employed to demonstrate the effectiveness of the proposed method. The fine-tuning results are shown in Figure 9.

In Figure 9, the fine-tuning results derived from the pruned model on the VisDrone and DIOR datasets are shown. The red dashed line represents the accuracy achieved by directly fine-tuning the pruned model, while the green solid line represents the accuracy obtained by fine-tuning the improved model with structural re-parameterization. From Figure 9a–c, it becomes evident that employing structural re-parameterization for fine-tuning the model achieved higher accuracy compared to the original fine-tuning model. Specifically, for the VisDrone dataset, when λ is equal to one and the pruning rate is 70%, the model with structural re-parameterization achieves a mAP@0.5 of 36.2% and a mAP@0.5:0.95 of 21.0%. This is 0.4% and 0.4% higher than the model without structural re-parameterization, respectively. Similarly, the DIOR dataset of Figure 9d–f demonstrates that utilizing structural re-parameterization for fine-tuning also outperforms the conventional method on the DIOR dataset. Notably, at λ equals 0.01 and a pruning rate of 60%, the introduced method achieves a mAP@0.5 of 79.8% and a mAP@0.5:0.95 of 58.6%. The mAP@0.5:0.95 shows a 0.5% enhancement compared to the conventional fine-tuning method. Drawing from the previously mentioned experimental outcomes, we can conclude that fine-tuning the pruned model through structural re-parameterization allows for deeper exploration of the feature extraction capabilities in lightweight models, thus improving the detection algorithm’s accuracy.

#### 4.3.4. The Comparison Results with Related Algorithms

To evaluate the proficiency of the proposed approach regarding model compression and detection precision, this study performs an in-depth comparative analysis between the proposed algorithms and leading lightweight detection algorithms on the Jetson AGX Xavier device.

In Table 5, it is demonstrated that MobileNetV3 maintains a certain advantage in terms of parameters, FLOPs, and feature extraction capability compared to other mainstream lightweight backbone networks. When replacing the YOLOv5m backbone network with a lightweight model, a significant decrease in parameters and FLOPs is attained, accompanied by a notable increase in FPS. Comparing MobileNetV3 with other lightweight backbone networks across parameters, FLOPs, FPS, and detection accuracy, it becomes evident that utilizing the MobileNetV3 model as the backbone network for YOLOv5m is the most suitable choice.

In Table 6, a comparison is made between the proposed algorithm and SSDLite, YOLOv3-MV2, YOLOv4-Tiny, YOLOv5s, YOLOv6n, YOLOv7-Tiny, YOLOXs, and YOLOv8n, from the perspective of parameters and FLOPs. The proposed method exhibits parameters of approximately 1–2 M and 6–7 G FLOPs, significantly lower than other mainstream lightweight detection algorithms, excluding SSDLite. Moreover, the proposed method demonstrates superior detection accuracy on both the VisDrone and DIOR datasets. Notably, it achieves mAP@0.5 values of 36.2% and 79.8% and mAP@0.5:0.95 values of 20.7% and 58.6% for the VisDrone and DIOR datasets, respectively. In terms of inference performance, our approach reaches a detection speed of 49 FPS on the VisDrone dataset and 44 FPS on the DIOR dataset. While the proposed algorithms lag behind YOLOv3-Tiny and YOLOv4-Tiny in terms of inference speed, they substantially outperform them in terms of detection accuracy.

**Figure 10:**
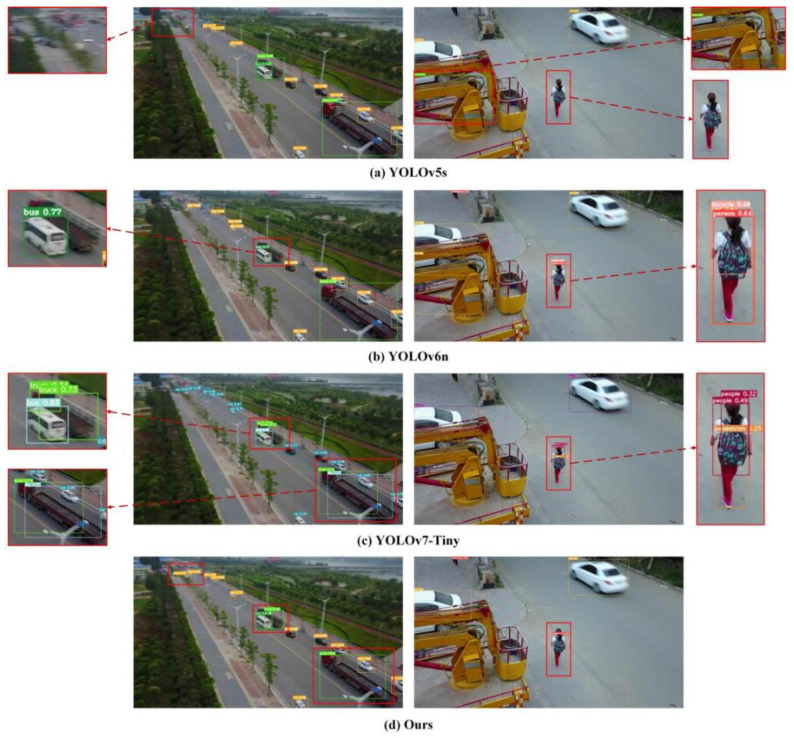
The detection results of mainstream lightweight detection algorithms in UAVs remote sensing scenes.

**Figure 11:**
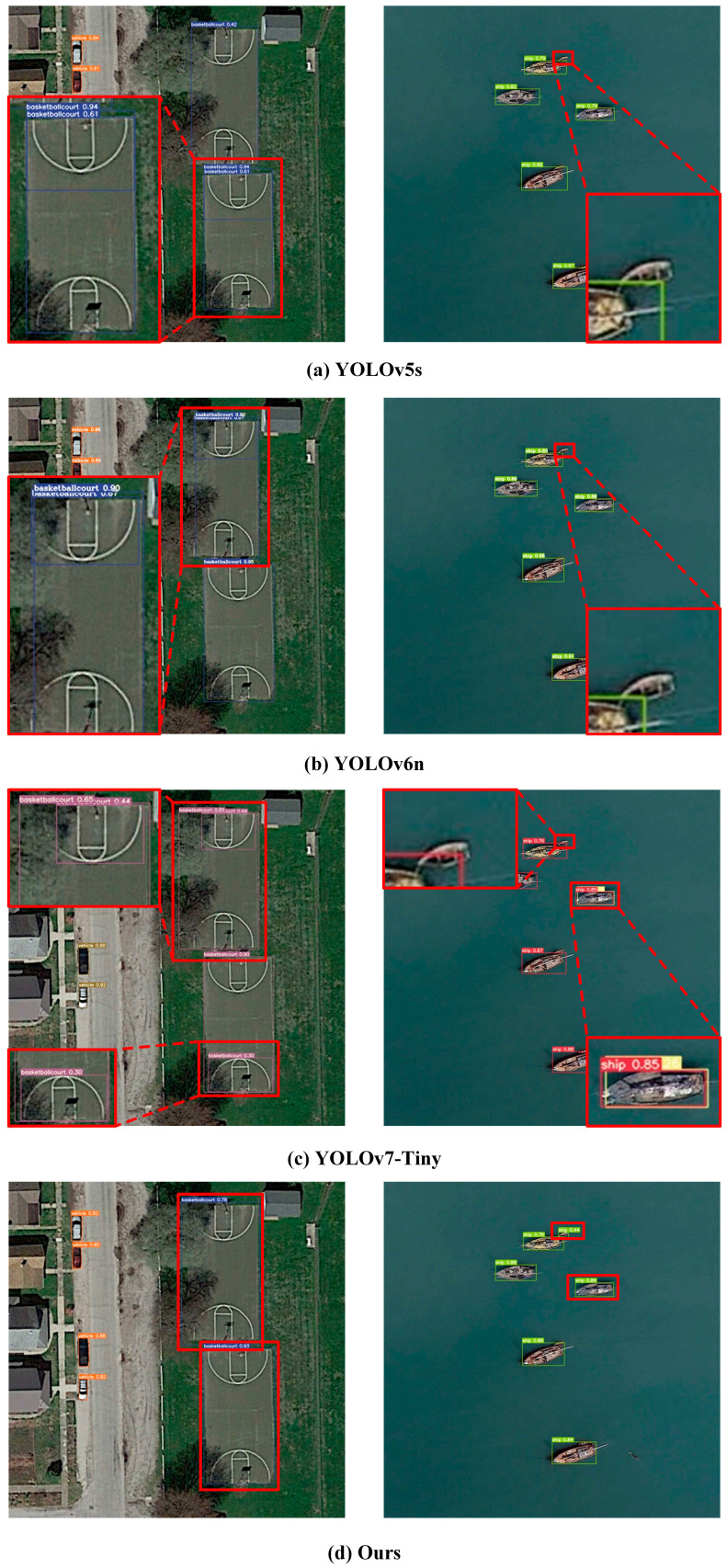
The detection results of mainstream lightweight detection algorithms in Satellite remote sensing scenes.

Figure 10 and Figure 11 show the performance contrast between the proposed algorithm and different mainstream lightweight detection methods in UAV remote sensing and Satellite remote sensing scenes.

Figure 10 presents the detection consequences of the algorithm in this article and different mainstream lightweight methods in UAV remote sensing scenes. In Figure 10a, the YOLOv5s algorithm exhibits poor detection capability for small objects, resulting in significant instances of missed detections. Similarly, both YOLOv6n and YOLOv7-Tiny demonstrate instances of redundant detections for the same object. Additionally, YOLOv6n shows a deficiency in detecting objects with prominent features. In contrast, the proposed algorithm outperforms these methods by effectively addressing these issues.

Figure 11 illustrates the detection performance comparison between select mainstream lightweight detection algorithms and the proposed algorithms in Satellite remote sensing scenes. As shown in Figure 11a–c, YOLOv5s, YOLOv6n, and YOLOv7-Tiny models still face the problem of missed identification for small targets in remote sensing scenes. Furthermore, these lightweight detection algorithms are prone to redundant detections for the same object (compared to Figure 10 and Figure 11, YOLOv7-Tiny is more prone to this problem).

## 5. Discussion

Table 4 demonstrates the performance impact of MobileNetV3, the ECA module, the FocalEIoU loss function, and the pruning fine-tuning method based on structural re-parameterization through ablation experiments. MobileNetV3 is chosen as the backbone network of YOLOv5m to lower the total algorithm coefficients, while the ECA module is integrated to augment the efficacy of feature detection within the core network structure. These optimizations reduce the computational cost of the model and improve the inference performance. However, the detection precision of the enhanced model is inferior to that of the baseline model. The reason is that the improved model has fewer parameters, which leads to insufficient learning capacity of the backbone network. In addition, the FocalEIoU loss function further enhances the detection capability of the YOLOv5 algorithm and compensates for the limitation of the CIoU in independently regressing width and height. The OPF refers to the original pruning fine-tuning method, while the RPF refers to the pruning fine-tuning method based on structural re-parameterization. The assessment results indicate that the RPF method can further enhance the performance of lightweight models, thus boosting their feature extraction capability.

Regarding the choice of MobileNetV3 as the backbone network for YOLOv5m, Table 5 shows its strong performance with respect to parameters, FLOPs, detection precision, and inference speed. In contrast, emerging networks like GhostNetV2, MobileOne, and FasterNet have lightweight versions that cannot achieve the same level of accomplishment as MobileNetV3 in practical applications.

By performing a comparative analysis of the evaluation results shown in Table 6, the proposed method demonstrates remarkable achievements in various aspects, including parameters, FLOPs, and other relevant metrics. Despite having lower FLOPs compared to SSDLite, the proposed method significantly outperforms it regarding identification precision metrics. On top of the UAV remote sensing scenes, the Satellite remote sensing scenes were also harnessed for examining the competence of the formulated procedure. The results, as shown in Figure 10 and Figure 11, provide evidence of the proposed method effectively addressing challenges related to missed detections and redundant detections, surpassing other mainstream lightweight detection algorithms. This corroborates the elevated performance and universality of the proposed method.

## 6. Conclusions

This study proposed a lightweight object method based on an optimized YOLOv5m algorithm, specifically designed for UAV remote sensing scenes, with a focus on edge computing devices carried by UAVs. The improved algorithm addresses the issue of imbalance between parameters, computational complexity, and detection precision in conventional object detection methods. To strengthen and optimize the attribute identification prowess of the lightweight algorithm, a structural re-parameterization-based pruning fine-tuning method is introduced. The MobileNetV3 architecture is employed as the backbone network for YOLOv5m by substituting the SE attention mechanism with the ECA module. Additionally, the FocalEIoU loss function is utilized for accurate bounding box regression and makes up for shortcomings of CIoU. Pruning is applied to compress the parameter and computational intricacy of the algorithm, while structural re-parameterization is used in the backbone network to further enhance the learning competence of the model.

The results of the experiment indicate that the enhanced algorithm achieves mAP@0.5 of 36.2% and 79.8%, as well as mAP@0.5:0.95 of 20.7% and 58.6% on the VisDrone and the DIOR datasets, respectively. Moreover, the inference speed on the Jetson AGX Xavier reaches 49 FPS and 44 FPS for the respective datasets. In comparison with other mainstream object detection methods, the improved algorithm exhibits advantages such as reduced parameters and FLOPs, enhanced identification accuracy, and accelerated processing velocity. Furthermore, the proposed algorithm holds potential for applications as an upstream method in tasks such as UAV ground localization and UAV search and rescue across diverse scenes.

## Figures and Tables

**Table 1:** Training and testing hardware devices.

Type	Training Server	Embedded Platform
Device name	DELL Precision R7920	Jetson AGX Xavier
CPU	Intel Xeon(R) Gold 6254 CPU @ 3.10 GHz × 72	64-bit 8-core CPU (ARMv8.2)
GPU	Quadro RTX 8000 48 G × 2	512-core Volta GPU with Tensor Cores
RAM	DELL 64G × 2	16 G

**Table 2:** The anchor box of the VisDrone and the DIOR dataset.

Dataset	Anchor Box								
VisDrone	5, 5	7, 11	15, 9	12, 19	29, 16	22, 31	54, 30	38, 55	91, 76
DIOR	10, 15	30, 12	15, 36	46, 37	55, 109	130, 127	119, 252	340, 208	323, 412

**Table 3:** The training hyperparameter.

Hyperparameter	Conventional Training	Sparse Training	Fine-Tuning Training
Optimizer	SGD	Adam	AdamW
Batch size	32	32	32
Input size	640 × 640	640 × 640	640 × 640
Mosaic	True	True	True
Initial learning rate	0.01	0.01	0.0032
Final learning rate	0.01	0.01	0.12
Warmup epochs	3	3	2
Warmup bias learning rate	0.1	0.1	0.05
Warmup momentum	0.8	0.8	0.5
Weight decay	0.0005	0.0005	0.00036
Momentum	0.937	0.937	0.843

**Table 4:** Ablation experiment results of algorithm improvements on Jetson AGX Xavier.

V3	ECA	FocalEIoU	OPF	RPF	Params (M)	FLOPs (G)	mAP@0.5 (%)	mAP@0.5:0.95 (%)	FPS (Frame/s)
					20.9	48.0	**38.1**/79.4	**21.8**/57.8	29/17
**✔**					11.9	20.0	33.5/79.1	18.3/56.6	37/27
**✔**	**✔**				10.4	20.0	34.1/79.4	18.7/56.6	38/27
**✔**	**✔**	**✔**			10.4	20.0	34.3/79.0	19.3/57.5	38/27
**✔**	**✔**	**✔**	**✔**		**1.2**/**1.5**	**6.2/6.5**	35.8/**79.8**	20.3/58.1	**49/44**
**✔**	**✔**	**✔**		**✔**			36.2/**79.8**	20.7/**58.6**	

The bold denotes the optimal value. V3 denotes the MobileNetV3 backbone network, ECA denotes the efficient channel attention mechanism, OPF denotes pruning and fine-tuning, and RPF denotes pruning and fine-tuning with structural re-parameterization. The left side of the slash denotes the test results on the VisDrone dataset, and the dexter portion denotes the experimental outcomes on the DIOR dataset.

**Table 5:** Comparison of performance across various lightweight networks on the Jetson AGX Xavier.

Backbone	Params (M)	FLOPs (G)	mAP@0.5 (%)	mAP@0.5:0.95 (%)	FPS (Frame/s)
GhostNetV1	12.7	20.0	28.9/77.0	15.2/53.6	25/25
GhostNetV2	18.7	22.2	32.6/76.4	17.4/52.2	22/19
ShuffleNetV2	**10.9**	**19.5**	30.4/77.2	16.2/54.4	33/26
MobileOne	17.3	32.8	**33.6**/77.5	**18.5**/54.7	32/19
FasterNet	11.6	22.3	33.1/76.5	17.9/53.4	**41/30**
MobileNetV3	11.9	20.0	33.5/**79.1**	18.3/**56.6**	37/27

The bold represents the optimal value. The left side of the slash denotes the test results on the VisDrone dataset, and the dexter portion denotes the experimental outcomes on the DIOR dataset.

**Table 6:** Performance comparison of different lightweight detectors on the Jetson AGX Xavier.

Method	Params (M)	FLOPs (G)	mAP@0.5 (%)	mAP@0.5:0.95 (%)	FPS (Frame/s)
SSDLite-MV2	3.2	**2.8**	20.0/66.9	10.4/42.9	27/26
YOLOv3-MV2	3.7	6.6	24.8/70.0	11.7/43.1	31/29
YOLOv4-Tiny	5.9	16.2	24.3/68.3	13.5/42.4	48/**49**
YOLOv5s	7.0	16.0	33.1/78.3	17.6/54.3	45/39
YOLOv6n	4.6	11.3	31.9/40.7	18.3/24.4	42/41
YOLOv7-Tiny	6.0	13.1	**37.1**/78.3	18.9/55.4	45/44
YOLOv8n	3.0	8.1	34.3/78.5	20.2/58.1	-
YOLOXs	8.9	13.3	32.7/75.7	17.9/49.7	25/25
**Ours**	**1.2**/**1.5**	6.2/6.5	36.2/**79.8**	**20.7**/**58.6**	**49**/44

The bold represents the optimal value. The left side of the slash denotes the test results on the VisDrone dataset, and the dexter portion denotes the experimental outcomes on the DIOR dataset.

## Data Availability

The authors will provide the raw data supporting this article’s conclusions upon request.
